# Singleton-based species names and fungal rarity: Does the number really matter?

**DOI:** 10.1186/s43008-023-00137-2

**Published:** 2024-03-20

**Authors:** Jonathan Cazabonne, Allison K. Walker, Jonathan Lesven, Danny Haelewaters

**Affiliations:** 1 Ecology Research Group of Abitibi RCM, Forest Research Institute, Université du Québec en Abitibi-Témiscamingue, Amos, QC J9T 2L8 Canada; 2 Centre for Forest Research, Université du Québec à Montréal, Montreal, QC H3C 3P8 Canada; 3 Department of Biology, Acadia University, Wolfville, NS B4P 2R6 Canada; 4 Laboratoire Chrono-Environnement, UMR 6249 CNRS, Université de Bourgogne Franche-Comté, 25000 Besançon, France; 5 Forest Research Institute, Université du Québec en Abitibi-Témiscamingue, Rouyn-Noranda, QC J9X 5E4 Canada; 6 Research Group Mycology, Department of Biology, Ghent University, 9000 Ghent, Belgium; 7 Faculty of Science, University of South Bohemia, 370 05 Ceske Budejovice, Czech Republic; 8 Department of Ecology and Evolutionary Biology, University of Colorado Boulder, Boulder, CO 80309 USA; 9 Biology Centre of the Czech Academy of Sciences, Institute of Entomology, 370 05 Ceske Budejovice, Czech Republic

**Keywords:** Integrative taxonomy, Morphology, Rarity, Sequencing, Singletons, Species descriptions

## Abstract

Fungi are among the least known organisms on earth, with an estimated number of species between 1.5 and 10 million. This number is expected to be refined, especially with increasing knowledge about microfungi in undersampled habitats and increasing amounts of data derived from environmental DNA sequencing. A significant proportion of newly generated sequences fail to match with already named species, and thus represent what has been referred to as fungal “dark taxa”. Due to the challenges associated with observing, identifying, and preserving sporophores, many macro- and microfungal species are only known from a single collection, specimen, isolate, and/or sequence—a singleton. Mycologists are consequently used to working with “rare” sequences and specimens. However, rarity and singleton phenomena lack consideration and valorization in fungal studies. In particular, the practice of publishing new fungal species names based on a single specimen remains a cause of debate. Here, we provide some elements of reflection on this issue in the light of the specificities of the fungal kingdom and global change context. If multiple independent sources of data support the existence of a new taxon, we encourage mycologists to proceed with formal description, irrespective of the number of specimens at hand. Although the description of singleton-based species may not be considered best practice, it does represent responsible science in the light of closing the Linnean biodiversity shortfall.

## INTRODUCTION

In most subfields of mycology, the issue of specimen rarity has always been a major concern, especially when it comes to patterns of diversity (Gange et al. 2019) and conservation (Molina et al. 2011). However, fundamental questions regarding the treatment of rarity of fungal specimens in taxonomy and ecology have yet to be answered. Lim et al. (2012) demonstrated that rare species are very common in taxonomy and that the phenomenon of rarity has been little considered. In particular, considerations regarding rarity apply to microscopic organisms of which the vast majority of species are rare, or at least rarely recorded. Microbial, and more specifically fungal, diversity records are generally dominated—in terms of abundance—by a small number of “common” species, suggesting that being common is not frequent in microbial groups, but “widely distributed” species of fungi often turn out to be species complexes following more rigorous analysis (e.g., Pringle et al. 2005; Haelewaters et al. 2018).

Some researchers prefer not to describe singletons (Lim et al. 2012), i.e., species names of which the description is based on a single specimen. Many mycologists indeed choose not to describe potential new species based solely on a single specimen as it may lack sufficient supporting material and thus may be of limited taxonomic value. Although the *International Code of Nomenclature for algae, fungi, and plants* or Shenzhen Code (hereafter referred to as the *Code*) does not prevent a specimen singleton from being designated a type (Turland et al. 2018), it is generally recommended, especially by reviewers, to await the acquisition of additional material when proposing a name based on a singleton (Aime et al. 2021). This practice is also frequently followed when collections or isolates of a potentially new species of fungi have been obtained, but the number of specimens nevertheless remains limited. The microscopic dimension of fungal diversity is also greatly subject to the presence of singletons as it contains most of the described and estimated fungal species (Hawksworth 2001). Molecular approaches such as the metabarcoding of environmental DNA (eDNA) have been a game changer in the study of fungal diversity (Ruppert et al. 2019) while introducing numerous biases and concerns regarding species known only from few or single environmental sequences.

While the description of new species based on singletons is sometimes regarded as a somewhat improper practice, a number of aspects merit more discussion in the light of the specificities of the fungal kingdom and knowledge shortfalls (Hortal et al. 2015; Haelewaters et al. 2024). Although fungi represent great ecological and taxonomic diversity and are globally distributed covering all terrestrial and aquatic ecosystems, they: (1) suffer from a lack of documentation and theoretical and practical knowledge, (2) pose challenges to observe and describe due to their cryptic nature, (3) represent considerable protection and conservation issues; and (4) regularly show local to global rarity with risks for extinction (Gaston 1998; Harnik et al. 2012; Hull et al. 2015; Aime et al. 2021; Mueller et al. 2022). Here, we address the implications of recognizing fungal species based on singletons in both field- and molecular-based fungal taxonomy.

The definition of what is a “singleton” varies in the literature. Some studies use this term to designate specimens encountered only once in their field surveys, or single collections that may be composed of numerous specimens. Other studies define singletons in a taxonomic context—as a species only known and described from a single specimen, while single molecular sequences are also frequently considered as singletons. Here, we apply the following definitions to avoid confusion:

1. Singleton-based species: a species that is known and described from a specimen, collection or isolate singleton.
2. Specimen singleton: a single specimen.
3. Collection singleton: a single collection of a given species, usually made in the field, consisting of numerous specimens collected at the same place and at the same time (a “gathering” in the sense of the *Code*).
4. Isolate singleton: a single isolate of a given species, originating from culturing.
5. Molecular singleton: a single molecular sequence characterizing a species either generated from DNA barcoding or eDNA metabarcoding procedures that, in the current *Code*, cannot be considered as the type of a species when considered alone.

When the term “singleton” is used in a general context, it represents all previous definitions without making specific distinctions among them.

## LIMITATIONS AND RISKS OF SINGLETON-BASED SPECIES

### Lack of natural variation

Depending on the fungal group, one specimen may not be representative of the whole range of variability of the species and displays limited information about intra- and interspecific variations—which may lead to misinterpretation of species attributes such as phenology (Aghayeva et al. 2022). It may be that a specimen singleton lacks the salient diagnostic characteristics that differentiate it from all its closely related species. For instance, in some specimen singletons analyzed by Hosaka et al. (2018) across collections in Japan, microscopic characteristics (i.e., basidia, cystidia) were absent, while for others the availability of spores was limited. Those distinguishing features vary from one group to another but generally involve different life-cycle stages, notably either sexual or asexual characteristics, or both if applicable.

Similar issues are encountered in collection and isolate singletons. Although one collection or culture often includes multiple sporophores and thus some variability, it may still be not sufficiently representative of the phenotypic plasticity typical for the species and lack diagnostic characteristics. A single preserved culture of a species may happen to lack sexual characteristics, as reported for *Colletotrichum psidii* (Weir et al. 2012). This renders it particularly difficult to link asexual and sexual morphs in a single classification system that considers specimen, collection, or isolate singletons.

Species descriptions are also preferred to be based on fully mature specimens since they usually bear well-developed structures. However, even when specimen singletons are fully mature, they do not allow for the analysis of intraspecific variability (morphology, genetics, geographic distribution, ecological preferences, etc.) or accessing information on different life stages. Some morphological characteristics may, however, be better observed in immature material. This is well illustrated in gasteroid fungi (Bowerman and Groves 1962). The description of species based on immature or overmature specimen singletons may complicate taxonomic work when it comes to comparing descriptions with that of other species—and may lead to the designation of epitypes (see *Ophiocordyceps unilateralis*, Evans et al. 2011; *Paurocotylis pila*, Dennis 1975).

### Material quantity, deterioration, and loss

Specimen, collection, and isolate singletons could face a particular risk of material deterioration, e.g., due to improper drying or preservation techniques in fungaria. Stakes associated with the loss of singleton material are much higher than when multiple well-preserved and representative specimens are available. The *Code* requires a physical specimen or permanently preserved metabolically inactive isolate to exist as type (Art. 8.1, Sect. 2, Chap. II), with some exceptions for illustrations of types prior to 1 January 2007 (Art. 40.4, Sect. 2, Chap. V) or concerning microfungi (Art. 40.5, Sect. 2, Chap. V). Material deterioration may also interfere with successful molecular protocols. Well-preserved material greatly benefits the value given to specimen singletons and their use as extended specimens (Lendemer et al. 2020; Antonelli et al. 2023). Fossil fungal species are a special case; for those, a type specimen is a strict requirement (*Code,* Art. 8.5). Fossil taxa, which are by default almost always specimen singletons, are also frequently damaged (Taylor et al. 2012; Perreau et al. 2021). Hosaka et al. (2018) found that many specimen singletons of “extinct” mushroom species in Japan were contaminated with molds and fragmented.

If holotype specimens are degraded (e.g., no more or low quantities of available DNA), DNA is highly fragmented (e.g., ancient specimens; Miller et al. 2022), or DNA is challenging to extract and amplify (e.g., melanized tissues in some species of cladosporioid fungi and *Laboulbeniales* microfungi; Moslem et al. 2010; Haelewaters et al. 2015), DNA extraction may be hindered or result in fragmented barcode sequences not suitable for species descriptions. As a consequence, specimen singletons are particularly susceptible to being described as species new to science solely based on morphology, which is not in accordance with best practices for the ideal of an integrative taxonomy. Morphological divergence is not always indicative of molecular divergence since the evolution of morphological characters may be faster than molecular characters and vice versa (Jargeat et al. 2010). Moreover, certain groups of fungi have few morphological characteristics, and some species may exhibit cryptic diversity or high phenotypic plasticity (e.g., Slepecky and Starmer 2009; Haelewaters et al. 2018; Hapuarachchi et al. 2019; Van Caenegem et al. 2023a). These observations highlight the need to combine morphological and molecular data whenever possible when dealing with singleton-based species—in line with using integrated methodologies to study fungal diversity (Cazabonne et al. 2022).

### Molecular singleton bias

One marker—notably the ITS region—can be sufficient to achieve resolution at the species level, hence its adoption as a universal barcode for fungi (Schoch et al. 2012). Many fungal species descriptions are indeed now being associated with a single ITS sequence obtained from the type, thus considered a molecular singleton. Fungal species based on both specimen singletons and molecular singletons are increasingly found in the literature (but see Aime et al. 2021). However, some groups of fungi may require more than one marker. One marker also goes against best practices of integrative taxonomic frameworks that use multiple loci for species delimitation methods (Cao et al. 2021; Chethana et al. 2021). Moreover, interspecific divergence in the ITS barcode can be lacking between two sister taxa (Kauserud 2023) whereas different copies of the ITS can be found in a single genome (Hibbett 2016; Paloi et al. 2022). Recently, Bradshaw et al. (2023) observed around 65% of 641 fungal species having intragenomic ITS variation. This is an important observation to consider when barcoding a single fungal specimen.

In fungal diversity studies based on both DNA barcoding and eDNA metabarcoding, molecular singletons are common and usually systematically removed from analyses to avoid biased interpretation and errors in sequencing. Molecular singletons derived from sequenced specimens are also often removed from macrofungal ecological analyses, even though these singletons represent already described fungal species and most of the collected specimens in the sampled area (Holec and Kučera 2020). Molecular singletons may artificially inflate diversity matrices and “species hypotheses” (Kõljalg et al. 2013) and result in non-asymptotic accumulation curves of species diversity (Sota et al. 2014). They contain a strongly elevated proportion of insertions compared with natural intra- and interspecific variation (Tedersoo et al. 2010; Sota et al. 2014). Hibbett et al. (2011) found that chimeric sequences are also common in environmental molecular singletons.

Metabarcoding of eDNA can fail to detect species present in situ or detect species that are not present in the analyzed samples (false positives) (Ficetola et al. 2016). Moreover, new species can be “created” by errors inherent to sequencing machines; we designate these here as “silico species”. Silico species are, by definition, created de novo and thus do not occur in nature. Although this phenomenon does exist, it has only been sparsely discussed in mycological literature. Further investigations are necessary to tackle challenges posed by false positive and silico species in fungal molecular studies, notably for the detection and distribution of molecular singletons in environmental samples.

Non-culturable microscopic fungal species known from a single sequence deserve specific taxonomic and systematic treatments. The issue of DNA-based typification of environmental samples has already been discussed by mycologists and is under active consideration (May and Redhead 2018; Nilsson et al. 2023). A committee looking into this is planned to report to the International Mycological Congress (IMC) nomenclature session in 2024 when a change could be approved.

### Singleton-based species threaten nomenclatural and taxonomic works

The full extent of taxonomic characters necessary to support the description of a new species is ideally gathered from multiple specimens from different developmental and life cycle stages and perhaps localities, where possible, and is complemented with multiple independent lines of support. Otherwise, the risks are to (1) either describe a novel species when there is none, or (2) not to describe a novel species when there is one. The distinctive characteristics associated with a specimen, collection, or isolate singleton can either be perceived as discriminating a new species or in other cases be considered as extending knowledge of the natural morphological variability of an already known species. With molecular singletons, biases related to sequencing errors can mislead mycologists and pass off simple genetic variations as evidence of a new species.

In case (1), new collections will be necessary to show broader morphological and molecular variability and recognize that a singleton-based species falls within the circumscription of an already described species, causing the name to be synonymized. In case (2), the new but unrecognized singleton-based species may find itself lost among different species (see Hawksworth and Rossman 1997 for related insights in phytopathogenic fungi). Moreover, the phenomenon of multiple fungal taxa grouped under one name disables efficient scientific communication (Ryberg and Nilsson 2018). Further screening and sequencing of fungarium collections may uncover those hidden singleton-based species. Still, this can lead to uphill taxonomic and nomenclatural work—especially if no other specimens, collections, or isolates are available to serve as lecto-, neo-, or epitypes.

## REASONS TO DESCRIBE AND PUBLISH SINGLETON-BASED SPECIES

### Specimen singletons as types are acceptable

The limits of action of fungal nomenclature are governed by the *Code* which continues to be improved (Turland et al. 2018; May et al. 2019). The *Code* does not prohibit the description of new fungal species based on specimen singletons. Alongside articles of the code related to typification, the examination of sufficient material to support species description is one informal requirement that should be checked by editors and reviewers for publication. Aime et al. (2021) discourage the publication of cryptic species based on a single collection without strong supporting evidence. As such, a fungal species can be published “with limited material but clear taxonomic novelty” (Seifert and Rossman 2010)—even though it is not in accordance with best practices—which de facto implicitly includes the case of single specimens.

### Many fungi are rare and only known from singletons

The phenomenon of rarity is predominant in the fungal kingdom. Fungal species based on specimen, collection, and isolate singletons are common, especially in understudied groups of fungi and from understudied areas of the world. For instance, species represented by collection and isolate singletons dominate corticioid collections in North American pine and spruce forests (Rosenthal et al. 2017), leaves in moist tropical forest of Central Panama (Arnold et al. 2000), the phyllosphere of temperate *Quercus macrocarpa* (Jumpponen and Jones 2009), and the romaine lettuce phylloplane (Haelewaters et al. 2021). Species of *Hermatomyces* (*Pleosporales*, *Hermatomycetaceae*) are often described based on single isolates or specimens collected from plant material (Koukol et al. 2018; Delgado et al. 2020). In some specific sampling locations, most macrofungi are known from a unique specimen in a single location (e.g., Malaysia; Mohammad et al. 2019). Some species are known from different locations only once (i.e., one mention per country; Haelewaters et al. 2024) and species that are presumed “extinct” are sometimes known only from a single type specimen in collections (e.g., Ogasawara Islands, Japan; Hosaka et al. 2018).

High-throughput sequencing technologies make it possible to feed public databases and discover new phylogenetic lineages (“dark taxa”; Lücking et al. 2021). Still, some species are known only by one environmental sequence. Baldrian et al. (2022) found that 2.5% of the 9.6 million singletons in their analysis had more than 97% shared identity with a fungal Species Hypothesis in UNITE. The numerous molecular singletons retrieved by Geml et al. (2009) suggest that there are still many species of *Lactarius* species awaiting discovery in Alaska. Molecular singletons may represent true or even undescribed species; however, they should be tackled with caution since many errors and biases are attributed to the sequencing process.

### Improving the taxonomy and phylogeny of poorly studied groups

Understudied fungal groups generally comprise challenging specimens to observe, collect, and identify in the field. This may be due in part to their small size, microscopic nature, interactions with plants (e.g., endophytes), insects (e.g., ectoparasitic microfungi), mosses, and lichens (bryophilous and lichenicolous fungi), as well as their surrounding habitat (e.g., soil and aquatic microfungi). Fungi of such groups are generally collected by experienced taxonomists during field expeditions, which are expensive, time-consuming, and thus relatively rare. Once collected, it is often difficult to re-collect. If such collections—many of which are collection singletons—are not formally described, their taxonomic information will be lost. As a result, the time interval between the collection and publication of these taxa, referred to as “shelf time” (Fontaine et al. 2012), increases and conservation efforts of the habitat they occur in will render incomplete.

For example, limited morphology coupled with molecular data was sufficient to formally describe the marine fungus *Lulworthia fundyensis* from a single specimen (Crous et al. 2022). Despite years of trying, authors were unable to get the fungus to reproduce sexually in culture or on wood bait (A.K. Walker unpubl.). And the monotypic genus *Globosphaeria* with type species *G. jamesii* was described on very scant material (only two ascomata on the squamules of the lichen *Normandina pulchella*) after extensive searches on the same host during several years failed to discover more material (Hawksworth 1990). Specimen, collection, and isolate singletons may harbor relevant data that may greatly benefit the scientific community. As an example, *Bimuria novae-zelandiae*, described based on an isolate singleton (Hawksworth et al. 1979), proved significant in illuminating *Dothideomycetes* phylogeny when sequenced (based on ribosomal DNA, Lumbsch and Lindemuth 2001; based on whole-genome scale data, Haridas et al. 2020). If possible, one should therefore describe collections for others to use in future work. Describing and appreciating the taxonomic value of singleton-based species contributes to acknowledging a reality that mycologists routinely encounter, thereby permitting them to address questions related to fungal rarity in the environment that should not be postponed against global change.

### Closing knowledge shortfalls while advancing conservation efforts

The description of a singleton-based fungal species is relevant for species of which formal description will assist in conservation efforts and legislation, for the fungus itself or for its potentially threatened associated organisms. The shelf time for a specimen singleton is pivotal since habitats might have been already altered by natural and anthropogenic factors. This issue is exacerbated in understudied fungal groups, which are characterized by knowledge gaps in species descriptions (Linnean shortfall), geographic distributions (Wallacean shortfall), most recent observations (Latimerian shortfall), and IUCN Red List assessments (Scottian shortfall) (Hortal et al. 2015; Haelewaters et al. 2024). The probability of re-collection could be greatly impacted with increasing shelf time, among others because of shifted phenology patterns. In *Colletotrichum*, many species known from a single occurrence or specimen have not been recorded afterwards despite sampling efforts, suggesting they might be endangered or extinct (Talhinhas and Baroncelli 2021). As such, specimen singletons may be representatives of species at risk for extinction.

As an example of how singletons can contribute to conservation efforts, Subramanian and Bhat (1987) described 21 new species and six new genera of hyphomycetes in the Silent Valley, Western Ghats, India. Only three of the newly described species were based on more than one collected specimen. Although fungi were just one of the dimensions of the intense surveys (also see Subramanian 2015), this cataloging of the rare fungal diversity of the region (the rarity being emphasized by the fact that most of the fungi were collected only once) played a key role in the protection of the Valley’s biodiversity. This was especially achieved by preventing the flooding of the Valley via stalling the construction of a dam project and founding a National Park.

### Considerations from other organismal groups

Species in other kingdoms represented by singletons are a well-known phenomenon. An estimated 30% of described invertebrate species are known from specimen singletons (Lim et al. 2012). Samples of southern African *Scarabaeidae* from Ahrens et al. (2016) included 49% singletons. In Ahrens et al.’s (2021) study of phytophagous scarab beetles in Sri Lanka, around 27% of all species were represented by a single specimen and 14 of the 27 morphospecies singletons were also molecular singletons. Conversely, it seems that plant species are rarely described based on singletons (Lim et al. 2012).

Descriptions of animal species based on a single specimen are accepted for the *International Code of Zoological Nomenclature* (Art. 61.1.2; International Commission on Zoological Nomenclature 1999), and there are no restrictions regarding the description of species from singletons (Kurina and Kirik 2021). As stated by Art. 73.1, a holotype can be based on a *single specimen*. In Art. 72.5, *an* animal (Art. 72.5.1) or *one* individual organism contained in a preparation for microscope examination (Art. 72.5.5) can be the name-bearing type of a species.

## CHALLENGES IN DEALING WITH SINGLETON-BASED SPECIES

The description of singleton-based species appears to be an acceptable practice in mycology that should be encouraged if multiple lines of evidence are assembled. However, most mycologists recognize that this is often not possible. We argue that descriptions of fungal species can be based on the holotype, even if there are not more reference specimens, collections, or isolates. Descriptions of singleton-based species (or specimens; e.g., Van Caenegem et al. 2023b) may be followed by field and molecular studies that add new data and expand the existing description as well as biogeographic, ecological, and genetic information.

If descriptions of specimen singletons are unavailable to the scientific community, researchers will not be able to link new collections to already recorded and collected species, whether or not formally named—publishing these will therefore facilitate further taxonomic works. Risks associated with publishing singleton-based species are thus far less than withholding relevant data from publication. Waiting for additional specimens should not be an obstacle to the advancement of fungal taxonomic knowledge. Although the aversion towards describing singletons appears to be overcome (Cheek et al. 2020), we identity the following challenges.

### Fungal singletons in a changing world filled with interactions

Fungal species described based on one specimen is a challenge in light of the “6th mass extinction”. The concern is twofold: to discover undescribed species and to explore the size of their natural populations, not solely of those in risk of extinction. Among the undiscovered threatened fungal species, there may be ecologically crucial singletons; their removal could have wider repercussions in species interaction networks and ecosystems. These facts should encourage building models to infer the current or potential extinction of fungal species known from only one specimen and one or very few locations (Roberts and Jaric 2020). A first avenue could be to quantify the proportion of currently known singleton-based fungal species in the literature, museum collections, and fungal data repositories. It is probable that many historical and well-known fungal species were originally described based on single specimens, collections, or isolates.

The probability of documenting a fungal species in interactions during field sampling is reduced when both the host and the associated fungus must be found occurring in the same environment at the same time. This concern is notably present in the fossil record of parasitic fungi where the host and the fungus do not occur simultaneously in preparations (Luo et al. 2023). The combined rarity of both fungal species and host complicates field observations and host screening, but even a single host specimen can bear multiple co-occurring fungal species (Hawksworth 2001). Some fungal groups may deserve specific attention as they are involved in interactions with other kingdoms subject to singleton-based species, such as insects (Novotný and Basset 2000; Kurina and Kirik 2021). Insects represent three-quarters of earth’s specific richness along with the fungi (Purvis and Hector 2000) and are involved in many poorly known insect–fungal interactions, which is likely to encompass a great pool of species known from singletons.

The number of macro- and microfungi described from singletons (or from just a few specimens) is likely to increase, knowing the documented effects of climate change on fungal phenology (Vogt-Schilb et al. 2022) and host range (Gange et al. 2011). Infection patterns of fungal ectoparasites are also impacted by changing environmental conditions (Szentiványi et al. 2019; Kaishian 2021). The emergence period of sporophores and the occurrence of fungal species on new hosts will be affected.

### Lack of expertise and resources

The expertise of experienced and trained mycologists is necessary when dealing with singleton-based species. To publish new species is highly dependent on the context, i.e., the state of preservation of the collection, fungal group, distinctiveness of the characteristics, sampling area, and number of worldwide fungal specialists working on that group. This requires the knowledge of mycologists who are aware of taxonomic issues in their group, e.g., to know if particular morphological differences or genetic variation can be considered indicators of a new species or genus. However, fungal taxonomy remains uncompetitive compared to other fields of mycology and is relatively poorly funded. Therefore, fungal taxonomy is underrepresented in academia and thus struggles to attract trainees. A recent study applied Red List methodologies to elucidate threats to insect taxonomists (Hochkirch et al. 2022). Many of their highlights are applicable to fungal taxonomists, including the shortfall of taxonomic expertise in many of the most species-diverse geographical areas. If no one is there to describe them, singleton-based species will be lost despite encouraged publication and recognition.

Current sampling efforts and resources may be insufficient to tackle the case of singleton-based fungal species. However, citizen science is still an untapped reservoir to generate data contributing to knowledge of fungal rarity. Specimen and collection singletons may come into the hands of amateur mycologists during mycological surveys and never be documented. Some singleton-based species might have even been documented in online databases (e.g., iNaturalist, MyCoPortal, MushroomObserver), or through social media, without the submitter or poster being aware that their posted material is the only representative of a potential species new to science. In recent efforts from the Fungal Diversity Survey in North America, citizen scientists are being encouraged through “Rare Challenges” (https://fundis.org/protect) to find local rare fungi. In addition, citizen science can contribute to a better knowledge of molecular singletons in environmental samples through projects aimed at collecting samples for eDNA studies. An ongoing example is FunLeaf (https://sisu.ut.ee/funleaf/about) that seeks to describe endophyte communities using leaves collected and sent by citizen scientists.

Despite being largely unused at present, museum collections represent treasure troves to fill knowledge gaps in biodiversity—including singletons (Miller et al. 2020; Card et al. 2021; Johnson et al. 2023). More than half of flowering plant species are estimated to have already been collected and stored in herbaria (Bebber et al. 2010). For fungi, many undescribed taxa are to be found in fungaria that may contain cryptic and new species “hidden” under old names or collections identified only to genus. However, non-digitized museum collections of fungi are poorly documented, with around 17% of known species held in culture collections and 55% in fungaria (Paton et al. 2020), and often suffer from curatorial neglect (Smith 2020). Fungaria in understudied geographic areas and poorly funded collections (e.g., those in the Global South) are of great interest since they are often representatives of the local funga and accommodate many singletons. Under-resourced collections face higher risks of material degradation (Paton et al. 2020) such as being consumed by arthropods or other pathogen attacks due to poor preservation conditions. They can sometimes stand as the only centralized institutional collections available for a wide geographical range and may house many actual and potential type specimens.

### Rarity in the light of fungal singletons

What is called *rarity* is highly specific to each group of organisms. It is difficult to distinguish between a lack of sampling efforts or true rarity (Bazzicalupo et al. 2022), notably in fungal groups in which increasing sampling efforts still return new species—a direct result of the Linnean shortfall (Haelewaters et al. 2024). In fact, many fungal species are probably more common than is estimated from collections. Thereby, fungal singletons and rarity are interlinked and intrinsically challenging, even more so when the definition of what is a species remains a matter of ongoing debate (*Glomeromycotina*; Bruns et al. 2018).

How singletons are perceived in fungal taxonomy is likely to be linked to our perception of biological rarity. For instance, for fungi only known from sporophores, it is difficult to determine when a species is rare. From a sporophore, one species would seem rare whereas most of its mycelium still resides in the environment. Moreover, sporophores are produced under specific environmental conditions; thus, a species may be invisible at the surface for several years, even decades, and then produce them in abundance in a specific year or under specific conditions. However, with global change, a rare fungal species today will probably not be equivalent to what a rare fungal species was in the past or will be in the future. Moreover, whether a recently alive and described fungal species is truly extinct would be arduous to determine given the rarity with which some species occur. As seen, rarity has various meanings within fungi, requiring adapted, universally accepted definitions (sensu Kaishian et al. 2022) of what a rare fungus is across fungal groups in evolving contexts—to allow for precise communication.

The place taken by the phenomena of fungal rarity may seem paradoxical given the immensity of the fungal kingdom; or that the large number of species, including their omnipresence and abundance, compensate for their rarity. Despite their diversity and resilience in the face of environmental disturbances, many known (and unknown) fungi may be threatened due to global change. The fungal kingdom is one of the least known groups of organisms in the scientific and societal sphere (but see Kuhar et al. 2018; Gonçalves et al. 2021; Palahí et al. 2022). Fungi challenge our current vision of what it means to be a rare species by giving pieces of evidence that being rare could be one of the most common ecological patterns in ecosystems.

## TOWARDS THE RECOGNITION OF FUNGI BEING SINGLE AND RARE

Singletons have traditionally been thought to be “poor science” (Cheek et al. 2020) because a single specimen from a single location cannot achieve complete taxonomic value (Cheek and Bridson 2019) and may remain therefore unpublished or published with missing data. However, singletons are very common across all kingdoms; thus the question “*Why are so many species based on a single specimen*” (fide Wells et al. 2019) also applies to organismal groups other than animals, including the fungi. The advent of molecular tools underlines the fact that fungal singletons are to be found in all ecosystems. Collecting or studying fungal singletons may cause a feeling of frustration since it is desirable to have more material to present when describing species (Aime et al. 2021). Nevertheless, a paradigm shift may be needed to address the challenges posed by global change; the high proportion of singletons from eDNA and in fungal inventories, suggests there is much unsampled fungal diversity and many new species remain to be discovered worldwide.

This manuscript calls for discussion, and we would be delighted to receive constructive feedback from the community. Fungal singletons go beyond the field of taxonomy and specialist mycologists; our lack of data on fungal singletons is symptomatic of our general lack of consideration of all fungi (Gonçalves et al. 2021). Even if any trade-off can be foreseen in the quality and relevance of species description because of insufficient material, scientists and the general public must work hand in hand to achieve reasonable goals of knowledge and recognition of singleton-based fungal species. We therefore encourage the community to value the smallest taxonomic or ecological data collected whether in peer-reviewed scientific journals or online databases for raw data and to put more effort into unravelling fungal singletons assuming many ecological functions, sometimes unsuspected, can be supported by them. To publish fungal singletons or not is no longer a question of best practice, but a question of necessity and responsibility at a time when the real rarity limiting our observations is not the specimens, but those who study them and the habitats that shelter them.

## Data Availability

Not applicable.

## Abbreviations

eDNA: Environmental DNA
ITS: Internal transcribed spacer

## References

[CR1] Aghayeva DN, Mustafabayli EH, Alimammadova AA, Yusifova YA (2022). Macrofungi of Goygol National Park and surrounding areas with special reference to medicinal species. Plant Fungal Res.

[CR2] Ahrens D, Ahyong ST, Ballerio A, Barclay MV, Eberle J, Espeland M, Huber BA, Mengual X, Pacheco TL, Peters RS, Rulik B, Vaz-de-Mello F, Wesener T, Krell FT (2021). Is it time to describe new species without diagnoses?—A comment on Sharkey et al. (2021). Zootaxa.

[CR3] Ahrens D, Fujisawa T, Krammer HJ, Eberle J, Fabrizi S, Vogler AP (2016). Rarity and incomplete sampling in DNA-based species delimitation. Syst Biol.

[CR4] Aime MC, Miller AN, Aoki T, Bensch K, Cai L, Crous PW, Hawksworth DL, Hyde KD, Kirk PM, Lücking R, May TW, Malosso E, Redhead SA, Rossman AY, Stadler M, Thines M, Yurkov AM, Zhang N, Schoch CL (2021). How to publish a new fungal species, or name, version 3.0. IMA Fungus.

[CR5] Antonelli A, Fry C, Smith RJ, Eden J, Govaerts RHA, Kersey P, Nic Lughadha E, Onstein RE, Simmonds MSJ, Zizka A, Ackerman JD, Adams VM, Ainsworth AM, Albouy C, Allen AP, Allen SP, Allio R, Auld TD, Bachman SP, Baker WJ, Barrett RL, Beaulieu JM, Bellot S, Black N, Boehnisch G, Bogarín D, Boyko JD, Brown MJM, Budden A, Bureš P, Butt N, Cabral A, Cai L, Cano JA, Chang Y, Charitonidou M, Chau JH, Cheek M, Chomicki G, Coiro M, Colli-Silva M, Condamine FL, Crayn DM, Cribb P, Cuervo-Robayo AP, Dahlberg A, Deklerck V, Denelle P, Dhanjal-Adams KL, Druzhinina I, Eiserhardt WL, Elliott TL, Enquist BJ, Escudero M, Espinosa-Ruiz S, Fay MF, Fernández M, Flanagan NS, Forest F, Fowler RM, Freiberg M, Gallagher RV, Gaya E, Gehrke B, Gelwick K, Grace OM, Granados Mendoza C, Grenié M, Groom QJ, Hackel J, Hagen ER, Hágsater E, Halley JM, Hu AQ, Jaramillo C, Kattge J, Keith DA, Kirk P, Kissling WD, Knapp S, Kreft H, Kuhnhäuser BG, Larridon I, Leão TCC, Leitch IJ, Liimatainen K, Lim JY, Lucas E, Lücking R, Luján M, Luo A, Magallón S, Maitner B, Márquez-Corro JI, Martín-Bravo S, Martins-Cunha K, Mashau AC, Mauad AV, Maurin O, Medina Lemos R, Merow C, Michelangeli FA, Mifsud JCO, Mikryukov V, Moat J, Monro AK, Muasya AM, Mueller GM, Muellner-Riehl AN, Nargar K, Negrão R, Nicolson N, Niskanen T, Oliveira Andrino C, Olmstead RG, Ondo I, Oses L, Parra-Sánchez E, Paton AJ, Pellicer J, Pellissier L, Pennington TD, Pérez-Escobar OA, Phillips C, Pironon S, Possingham H, Prance G, Przelomska NAS, Ramírez-Barahona SA, Renner SS, Rincon M, Rivers MC, Rojas Andrés BM, RomeroSoler KJ, Roque N, Rzedowski J, Sanmartín I, Santamaría-Aguilar D, Schellenberger Costa D, Serpell E, Seyfullah LJ, Shah T, Shen X, Silvestro D, Simpson DA, Šmarda P, Šmerda J, Smidt E, Smith SA, Solano-Gomez R, Sothers C, Soto Gomez M, Spalink D, Sperotto P, Sun M, Suz LM, Svenning JC, Taylor A, Tedersoo L, Tietje M, Trekels M, Tremblay RL, Turner R, Vasconcelos T, Veselý P, Villanueva BS, Villaverde T, Vorontsova MS, Walker BE, Wang Z, Watson M, Weigelt P, Wenk EH, Westrip JRS, Wilkinson T, Willett SD, Wilson KL, Winter M, Wirth C, Wölke FJR, Wright IJ, Zedek F, Zhigila DA, Zimmermann NE, Zuluaga A, Zuntini AR (2023) State of the world’s plants and fungi 2023. Royal Botanic Gardens, Kew. 10.34885/wnwn-6s63

[CR6] Arnold AE, Maynard Z, Gilbert GS, Coley PD, Kursar TA (2000). Are tropical fungal endophytes hyperdiverse?. Ecol Lett.

[CR7] Baldrian P, Větrovský T, Lepinay C, Kohout P (2022). High-throughput sequencing view on the magnitude of global fungal diversity. Fungal Divers.

[CR8] Bazzicalupo A, Gonçalves SC, Hébert R, Jakob S, Justo A, Kernaghan G, Lebeuf R, Malloch B, Thorn RG, Walker AK (2022). Macrofungal conservation in Canada and target species for assessment: a starting point. FACETS.

[CR9] Bebber DP, Carine MA, Wood JR, Wortley AH, Harris DJ, Prance GT, Davidse G, Paige J, Pennington TD, Robson NKB, Scotland R (2010). Herbaria are a major frontier for species discovery. Proc Natl Acad Sci.

[CR10] Bowerman CA, Groves JW (1962). Notes on fungi from northern Canada: V. Gasteromycetes. Can J Bot.

[CR11] Bradshaw MJ, Aime MC, Rokas A, Maust A, Moparthi S, Jellings K, Pane AM, Hendricks D, Pandey B, Li Y, Pfister DH (2023). Extensive intragenomic variation in the internal transcribed spacer region of fungi. iScience.

[CR12] Bruns TD, Corradi N, Redecker D, Taylor JW, Öpik M (2018). *Glomeromycotina*: What is a species and why should we care?. New Phytol.

[CR13] Cao B, Haelewaters D, Schoutteten N, Begerow D, Boekhout T, Giachini AJ, Gorjón SP, Gunde-Cimerman N, Hyde KD, Kemler M, Li GJ, Liu DM, Liu XZ, Nuytinck J, Papp V, Savchenko A, Savchenko K, Tedersoo L, Theelen B, Thines M, Tomšovský M, Toome-Heller M, Urón JP, Verbeken A, Vizzini A, Yurkov AM, Zamora JC, Zhao RL (2021). Delimiting species in *Basidiomycota*: a review. Fungal Divers.

[CR14] Card DC, Shapiro B, Giribet G, Moritz C, Edwards SV (2021). Museum genomics. Annu Rev Genet.

[CR15] Cazabonne J, Bartrop L, Dierickx G, Gafforov Y, Hofmann TA, Martin TE, Piepenbring M, Rivas-Ferreiro M, Haelewaters D (2022). Molecular-based diversity studies and field surveys are not mutually exclusive: on the importance of integrated methodologies in mycological research. Front Fungal Biol.

[CR16] Cheek M, Bridson D (2019). Three new threatened *Keetia* species (*Rubiaceae*-*Vanguerieae*), from the forests of the Eastern Arc Mountains, Tanzania. Gard Bull Singapore.

[CR17] Cheek M, Nic Lughadha E, Kirk P, Lindon H, Carretero J, Looney B, Douglas B, Haelewaters D, Gaya E, Llewellyn T, Ainsworth AM, Gafforov Y, Hyde K, Crous P, Hughes M, Walker BE, Forzza RC, Wong KM, Niskanen T (2020). New scientific discoveries: plants and fungi. Plants People Planet.

[CR18] Chethana TKW, Manawasinghe IS, Hurdeal VG, Bhunjun CS, Appadoo MA, Gentekaki E, Raspé O, Promputtha I, Hyde KD (2021). What are fungal species and how to delineate them?. Fungal Divers.

[CR200] Crous PW, Boers J, Holdom D, Osieck ER, Steinrucken TV, Tan YP, Vitelli JS, Shivas RG, Barrett M, Boxshall AG, Broadbridge J, Larsson E, Lebel T, Pinruan U, Sommai S, Alvarado P, Bonito G, Decock CA, De la Peña-Lastra S, Delgado G, Houbraken J, Maciá-Vicente JG, Raja HA, Rigueiro-Rodríguez A, Rodríguez A, Wingfield MJ, Adams SJ, Akulov A, AL-Hidmi T, Antonín V, Arauzo S, Arenas F, Armada F, Aylward J, Bellanger JM, Berraf-Tebbal A, Bidaud A, Boccardo F, Cabero J, Calledda F, Corriol G, Crane JL, Dearnaley JDW, Dima B, Dovana F, Eichmeier A, Esteve-Raventós F, Fine M, Ganzert L, García D, Torres-Garcia D, Gené J, Gutiérrez A, Iglesias P, Istel Ł, Jangsantear P, Jansen GM, Jeppson M, Karun NC, Karich A, Khamsuntorn P, Kokkonen K, Kolarík M, Kubátová A, Labuda R, Lagashetti AC, Lifshitz N, Linde C, Loizides M, Luangsa-ard JJ, Lueangjaroenkit P, Mahadevakumar S, Mahamedi AE, Malloch DW, Marincowitz S, Mateos A, Moreau PA, Miller AN, Molia A, Morte A, Navarro-Ródenas A, Nebesářová J, Nigrone E, Nuthan BR, Oberlies NH, Pepori AL, Rämä T, Rapley D, Reschke K, Robicheau BM, Roets F, Roux J, Saavedra M, Sakolrak B, Santini A, Ševčíková H, Singh PN, Singh SK, Somrithipol S, Spetik M, Sridhar KR, Starink-Willemse M, Taylor VA, van Iperen AL, Vauras J, Walker AK, Wingfield BD, Yarden O, Cooke AW, Manners AG, Pegg KG, Groenewald JZ (2022). Fungal Planet description sheets: 1383–1435. Persoonia.

[CR19] Delgado G, Koukol O, Heredia G, Piepenbring M (2020). Texas microfungi: *Hermatomyces amphisporus* (*Pleosporales*, *Dothideomycetes*) revisited. Czech Mycol.

[CR20] Dennis RWG (1975). New or interesting British microfungi, III. Kew Bull.

[CR21] Evans HC, Elliot SL, Hughes DP (2011). Hidden diversity behind the zombie-ant fungus *Ophiocordyceps unilateralis*: four new species described from carpenter ants in Minas Gerais, Brazil. PLoS ONE.

[CR22] Ficetola GF, Taberlet P, Coissac E (2016). How to limit false positives in environmental DNA and metabarcoding?. Mol Ecol Resour.

[CR23] Fontaine B, Perrard A, Bouchet P (2012). 21 years of shelf life between discovery and description of new species. Curr Biol.

[CR24] Gange AC, Allen LP, Nussbaumer A, Gange EG, Andrew C, Egli S, Senn-Irlet B, Boddy L (2019). Multiscale patterns of rarity in fungi, inferred from fruiting records. Glob Ecol Biogeogr.

[CR25] Gange AC, Gange EG, Mohammad AB, Boddy L (2011). Host shifts in fungi caused by climate change?. Fungal Ecol.

[CR26] Gaston K (1998). Rarity as double jeopardy. Nature.

[CR27] Geml J, Laursen GA, Timling I, McFarland JM, Booth MG, Lennon N, Nusbaum C, Taylor DL (2009). Molecular phylogenetic biodiversity assessment of arctic and boreal ectomycorrhizal *Lactarius* Pers. (*Russulales*; *Basidiomycota*) in Alaska, based on soil and sporocarp DNA. Mol Ecol.

[CR28] Gonçalves SC, Haelewaters D, Furci G, Mueller GM (2021). Include all fungi in biodiversity goals. Science.

[CR29] Haelewaters D, De Kesel A, Pfister DH (2018). Integrative taxonomy reveals hidden species within a common fungal parasite of ladybirds. Sci Rep.

[CR30] Haelewaters D, Gorczak M, Pfliegler WP, Tartally A, Tischer M, Wrzosek M, Pfister DH (2015). Bringing *Laboulbeniales* into the 21st century: enhanced techniques for extraction and PCR amplification of DNA from minute ectoparasitic fungi. IMA Fungus.

[CR31] Haelewaters D, Matthews TJ, Wayman JP, Cazabonne J, Heyman F, Quandt CA, Martin TE (2024). Biological knowledge shortfalls impede conservation efforts in poorly studied taxa—a case study of *Laboulbeniomycetes*. J Biogeogr.

[CR32] Haelewaters D, Urbina H, Brown S, Newerth-Henson S, Aime MC (2021). Isolation and molecular characterization of the romaine lettuce phylloplane mycobiome. J Fungi.

[CR33] Hapuarachchi KK, Karunarathna SC, McKenzie EHC, Wu XL, Kakumyan P, Hyde KD, Wen TC (2019). High phenotypic plasticity of *Ganoderma sinense* (*Ganodermataceae*, *Polyporales*) in China. Asian J Mycol.

[CR34] Haridas S, Albert R, Binder M, Bloem J, LaButti K, Salamov A, Andreopoulos B, Baker SE, Barry K, Bills G, Bluhm BH, Cannon C, Castanera R, Culley DE, Daum C, Ezra D, González JB, Henrissat B, Kuo A, Liang C, Lipzen A, Lutzoni F, Magnuson J, Mondo SJ, Nolan M, Ohm RA, Pangilinan J, Park HJ, Ramírez L, Alfaro M, Sun H, Tritt A, Yoshinaga Y, Zwiers LH, Turgeon BG, Goodwin SB, Spatafora JW, Crous PW, Grigoriev IV (2020). 101 *Dothideomycetes* genomes: a test case for predicting lifestyles and emergence of pathogens. Stud Mycol.

[CR35] Harnik PG, Simpson C, Payne JL (2012). Long-term differences in extinction risk among the seven forms of rarity. Proc R Soc B Biol Sci.

[CR36] Hawksworth DL (1990). *Globosphaeria*, a remarkable new pyrenomycete on *Normandina* from Tasmania. Lichenologist.

[CR37] Hawksworth DL (2001). The magnitude of fungal diversity: the 1.5 million species estimate revisited. Mycol Res.

[CR38] Hawksworth DL, Yen CC, Sheridan JE (1979). *Bimuria novae-zelandiae*, gen. et sp. Nov., a remarkable ascomycete from a New Zealand barley field. N Z J Bot.

[CR39] Hibbett D (2016). The invisible dimension of fungal diversity. Science.

[CR40] Hibbett DS, Ohman A, Glotzer D, Nuhn M, Kirk P, Nilsson RH (2011). Progress in molecular and morphological taxon discovery in Fungi and options for formal classification of environmental sequences. Fungal Biol Rev.

[CR41] Hochkirch A, Casino A, Penev L, Allen D, Tilley L, Georgiev T, Gospodinov K, Barov B (2022). European Red List of insect taxonomists. Publication Office of the European Union, Luxembourg.

[CR42] Holec J, Kučera T (2020). Richness and composition of macrofungi on large decaying trees in a Central European old-growth forest: a case study on silver fir (*Abies alba*). Mycol Prog.

[CR43] Hortal J, de Bello F, Diniz-Filho JAF, Lewinsohn TM, Lobo JM, Ladle RJ (2015). Seven shortfalls that beset large-scale knowledge of biodiversity. Annu Rev Ecol Evol Syst.

[CR44] Hosaka K, Kobayashi T, Castellano MA, Orihara T (2018). The status of voucher specimens of mushroom species thought to be extinct from Japan. Bull Natl Mus Nat Sci Ser B.

[CR45] Hull PM, Darroch SA, Erwin DH (2015). Rarity in mass extinctions and the future of ecosystems. Nature.

[CR46] International Commission on Zoological Nomenclature (1999) *International Code of Zoological Nomenclature*, 4th ed. International Trust for Zoological Nomenclature, London, I–XXIX, 1–306. https://www.iczn.org/the-code/the-code-online/10.3897/zookeys.931.51583 PMC7205856 32405237

[CR47] Jargeat P, Martos F, Carriconde F, Gryta H, Moreau PA, Gardes M (2010). Phylogenetic species delimitation in ectomycorrhizal fungi and implications for barcoding: the case of the *Tricholoma scalpturatum* complex (*Basidiomycota*). Mol Ecol.

[CR48] Johnson KR, Owens IF, Global Collection Group (2023) A global approach for natural history museum collections. Science 379(6638):1192–1194. 10.1126/science.adf6434 10.1126/science.adf6434 36952410

[CR49] Jumpponen A, Jones KL (2009). Massively parallel 454 sequencing indicates hyperdiverse fungal communities in temperate *Quercus macrocarpa* phyllosphere. New Phytol.

[CR50] Kaishian PJ (2021). Insects and their *Laboulbeniales* (*Ascomycota*, *Fungi*) of Lake Eustis and Emeralda Marsh Conservation Area: A case study on urbanization and diversity. Ecol Evol.

[CR51] Kaishian P, Lubbers M, de Groot MD, Schilthuizen M, Haelewaters D (2022) Definitions of parasites and pathogens through time. Authorea. 10.22541/au.165712662.22738369/v1

[CR52] Kauserud H (2023). ITS alchemy: on the use of ITS as a DNA marker in fungal ecology. Fungal Ecol.

[CR53] Kõljalg U, Nilsson RH, Abarenkov K, Tedersoo L, Taylor AF, Bahram M, Bates ST, Bruns TD, Bengtsson-Palme J, Callaghan TM, Douglas B (2013). Towards a unified paradigm for sequence-based identification of fungi. Mol Ecol.

[CR54] Koukol O, Delgado G, Hofmann TA, Piepenbring M (2018). Panama, a hot spot for *Hermatomyces* (*Hermatomycetaceae*, *Pleosporales*) with five new species, and a critical synopsis of the genus. IMA Fungus.

[CR55] Kuhar F, Furci G, Drechsler-Santos ER, Pfister DH (2018). Delimitation of Funga as a valid term for the diversity of fungal communities: the Fauna, Flora & Funga proposal (FF&F). IMA Fungus.

[CR56] Kurina O, Kirik H (2021). Every single specimen counts: a new *Docosia* Winnertz (*Diptera*: *Mycetophilidae*) species described from a singleton. Insects.

[CR57] Lendemer J, Thiers B, Monfils AK, Zaspel J, Ellwood ER, Bentley A, LeVan K, Bates J, Jennings D, Contreras D, Lagomarsino L, Mabee P, Ford LS, Guralnick R, Gropp RE, Revelez M, Cobb N, Seltmann K, Aime MC (2020). The extended specimen network: a strategy to enhance US biodiversity collections, promote research and education. Bioscience.

[CR58] Lim GS, Balke M, Meier R (2012). Determining species boundaries in a world full of rarity: singletons, species delimitation methods. Syst Biol.

[CR59] Lücking R, Aime MC, Robbertse B, Miller AN, Aoki T, Ariyawansa HA, Cardinali G, Crous PW, Druzhinina IS, Geiser DM, Hawksworth DL, Hyde KD, Irinyi L, Jeewon R, Johnston PR, Kirk PM, Malosso E, May TW, Meyer W, Nilsson HR, Öpik M, Robert V, Stadler M, Thines M, Vu D, Yurkov AM, Zhang N, Schoch CL (2021). Fungal taxonomy and sequence-based nomenclature. Nat Microbiol.

[CR60] Lumbsch HT, Lindemuth R (2001). Major lineages of *Dothideomycetes* (*Ascomycota*) inferred from SSU and LSU rDNA sequences. Mycol Res.

[CR61] Luo C, Haelewaters D, Krings M (2023) Fossils of parasitic fungi. Authorea. 10.22541/au.166696714.49994889/v2

[CR62] May TW, Redhead SA (2018). Synopsis of proposals on fungal nomenclature: a review of the proposals concerning Chapter F of the *International Code of Nomenclature for algae, fungi, and plants* submitted to the XI International Mycological Congress, 2018. IMA Fungus.

[CR63] May TW, Redhead SA, Bensch K, Hawksworth DL, Lendemer J, Lombard L, Turland NJ (2019). Chapter F of the International Code of Nomenclature for algae, fungi, and plants as approved by the 11th International Mycological Congress, San Juan, Puerto Rico, July 2018. IMA Fungus.

[CR64] Miller SE, Barrow LN, Ehlman SM, Goodheart JA, Greiman SE, Lutz HL, Misiewicz TM, Smith SM, Tan M, Thawley CJ, Cook JA, Light JE (2020). Building natural history collections for the twenty-first century and beyond. Bioscience.

[CR65] Miller AN, Karakehian J, Raudabaugh DB (2022). Next-generation sequencing of ancient and recent fungarium specimens. J Fungi.

[CR66] Mohammad A, Yee LS, Sawawi N (2019). Macrofungi of Pulau Sibu, Johor: a preliminary study. Malay Nat J.

[CR67] Molina R, Horton TR, Trappe JM, Marcot BG (2011). Addressing uncertainty: how to conserve and manage rare or little-known fungi. Fungal Ecol.

[CR68] Moslem MA, Bahkali AH, Abd-Elsalam KA, de Wit PJGM (2010). An efficient method for DNA extraction from cladosporioid fungi. Genet Mol Res.

[CR69] Mueller GM, Cunha KM, May TW, Allen JL, Westrip JR, Canteiro C, Costa-Rezende DH, Drechsler-Santos ER, Vasco-Palacios AM, Ainsworth AM, Alves-Silva G, Bungartz F, Chandler A, Gonçalves SC, Krisai-Greilhuber I, Iršėnaitė R, Jordal JB, Kosmann T, Lendemer J, McMullin RT, Mešić A, Motato-Vásquez V, Ohmura Y, Næsborg RR, Perini C, Saar I, Simijaca D, Yahr R, Dahlberg A (2022). What do the first 597 Global Fungal Red List assessments tell us about the threat status of fungi?. Diversity.

[CR70] Nilsson RH, Ryberg M, Wurzbacher C, Tedersoo L, Anslan S, Põlme S, Spirin V, Mikryukov V, Svantesson S, Hartmann M, Lennartsdotter C, Belford P, Khomich M, Retter A, Corcoll N, Martinez DG, Jansson T, Ghobad-Nejhad M, Vu D, Sanchez-Garcia M, Kristiansson E, Abarenkov K (2023) How, not if, is the question mycologists should be asking about DNA-based typification. MycoKeys 96:143–157. 10.3897/mycokeys.96.102669 10.3897/mycokeys.96.102669 PMC10194844 37214179

[CR71] Novotný V, Basset Y (2000). Rare species in communities of tropical insect herbivores: pondering the mystery of singletons. Oikos.

[CR72] Palahí M, Kiers T, Sheldrake M, Furci G, Nasi R, César RG (2022). Republish: open letter on the crucial role of fungi in preserving and enhancing biodiversity. Stud Fungi.

[CR73] Paloi S, Luangsa-Ard JJ, Mhuantong W, Stadler M, Kobmoo N (2022). Intragenomic variation in nuclear ribosomal markers and its implication in species delimitation, identification and barcoding in *Fungi*. Fungal Biol Rev.

[CR74] Paton A, Antonelli A, Carine M, Forzza RC, Davies N, Demissew S, Dröge G, Fulcher T, Grall A, Holstein N, Jones M (2020). Plant and fungal collections: current status, future perspectives. Plants People Planet.

[CR75] Perreau M, Haelewaters D, Tafforeau P (2021). A parasitic coevolution since the Miocene revealed by phase-contrast synchrotron X-ray microtomography and the study of natural history collections. Sci Rep.

[CR76] Pringle A, Baker DM, Platt JL, Wares JP, Latgé JP, Taylor JW (2005). Cryptic speciation in the cosmopolitan and clonal human pathogenic fungus *Aspergillus fumigatus*. Evolution.

[CR77] Purvis A, Hector A (2000). Getting the measure of biodiversity. Nature.

[CR78] Roberts DL, Jarić I (2020). Inferring the extinction of species known only from a single specimen. Oryx.

[CR79] Rosenthal LM, Larsson KH, Branco S, Chung JA, Glassman SI, Liao HL, Peay KG, Smith DP, Talbot JM, Taylor JW, Vellinga EC (2017). Survey of corticioid fungi in North American pinaceous forests reveals hyperdiversity, underpopulated sequence databases, and species that are potentially ectomycorrhizal. Mycologia.

[CR80] Ruppert KM, Kline RJ, Rahman MS (2019). Past, present, and future perspectives of environmental DNA (eDNA) metabarcoding: A systematic review in methods, monitoring, and applications of global eDNA. Glob Ecol Conserv.

[CR81] Ryberg M, Nilsson RH (2018). New light on names and naming of dark taxa. MycoKeys.

[CR82] Schoch CL, Seifert KA, Huhndorf S, Robert V, Spouge JL, Levesque CA, Chen W, Fungal Barcoding Consortium (2012). Nuclear ribosomal internal transcribed spacer (ITS) region as a universal DNA barcode marker for *Fungi*. Proc Natl Acad Sci USA.

[CR83] Seifert KA, Rossman AY (2010). How to describe a new fungal species. IMA Fungus.

[CR84] Slepecky RA, Starmer WT (2009). Phenotypic plasticity in fungi: a review with observations on *Aureobasidium pullulans*. Mycologia.

[CR85] Smith N (2020). Minority taxa, marginalised collections: A focus on fungi. J Nat Sci Collect.

[CR86] Sota T, Kagata H, Ando Y, Utsumi S, Osono T (2014) Metagenomic approach yields insights into fungal diversity and functioning. In: Sota T, Kagata H, Ando Y, Utsumi S, Osono T (eds) Species diversity and community structure. Springer, Tokyo, pp 1–23. 10.1007/978-4-431-54261-2_1

[CR87] Subramanian CV (2015). The pursuit of mycology in the tropics: recollections. Kavaka.

[CR88] Subramanian CV, Bhat DJ (1987). Hyphomycetes from South India I. Some New Taxa Kavaka.

[CR89] Szentiványi T, Haelewaters D, Rádai Z, Mizsei E, Pfliegler WP, Báthori F, Tartally A, Christe P, Glaizot O (2019). Climatic effects on the distribution of ant-and bat fly-associated fungal ectoparasites (*Ascomycota*, *Laboulbeniales*). Fungal Ecol.

[CR90] Talhinhas P, Baroncelli R (2021). *Colletotrichum* species and complexes: geographic distribution, host range and conservation status. Fungal Divers.

[CR91] Taylor TN, Krings M, Galtier J, Dotzler N (2012). Fungal endophytes in *Astromyelon*-type (*Sphenophyta*, *Equisetales*, *Calamitaceae*) roots from the Upper Pennsylvanian of France. Rev Palaeobot Palynol.

[CR92] Tedersoo L, Nilsson RH, Abarenkov K, Jairus T, Sadam A, Saar I, Bahram M, Bechem E, Chuyong G, Kõljalg U (2010). 454 pyrosequencing and Sanger sequencing of tropical mycorrhizal fungi. New Phytol.

[CR93] Turland N, Wiersema JH, Barrie FR, Greuter W, Hawksworth DL, Herendeen PS, Knapp S, Kusber WH, Li DZ, Marhold K, May TW, McNeill J, Monro AM, Prado J, Price MJ, Smith GF (2018) International code of nomenclature for algae, fungi, and plants (Shenzhen code) adopted by the nineteenth international botanical congress Shenzhen, China, July 2017. Regnum Vegetabile 159. Koeltz Botanical Books, Glashütten. 10.12705/Code.2018

[CR94] Van Caenegem W, Blondelle A, Dumolein I, Santamaria B, Dick CW, Hiller T, Liu J, Quandt CA, Villarreal Saucedo RV, Verbeken A, Haelewaters D (2023). Five new species of *Gloeandromyces* (*Fungi*, *Laboulbeniales*) from tropical American bat flies (*Diptera*, *Streblidae*), revealed by morphology and phylogenetic reconstruction. Mycologia.

[CR95] Van Caenegem W, Ceryngier P, Romanowski J, Pfister DH, Haelewaters D (2023). *Hesperomyces* (*Fungi*, *Ascomycota*) associated with *Hyperaspis* ladybirds (*Coleoptera*, *Coccinellidae*): rethinking host specificity. Front Fungal Biol.

[CR96] Vogt-Schilb H, Richard F, Malaval JC, Rapior S, Fons F, Bourgade V, Schatz B, Buentgen U, Moreau PA (2022). Climate-induced long-term changes in the phenology of Mediterranean fungi. Fungal Ecol.

[CR97] Weir BS, Johnston PR, Damm U (2012). The *Colletotrichum gloeosporioides* species complex. Stud Mycol.

[CR98] Wells A, Johanson KA, Dostine P (2019). Why are so many species based on a single specimen?. Zoosymposia.

